# Study of the Relationships between the Spatial Extent of Surface Urban Heat Islands and Urban Characteristic Factors Based on Landsat ETM+ Data

**DOI:** 10.3390/s8117453

**Published:** 2008-11-20

**Authors:** Jinqu Zhang, Yunpeng Wang

**Affiliations:** 1 State Key Laboratory of Organic Geochemistry, Guangzhou Institute of Geochemistry, Chinese Academy of Sciences, P. O. Box 1131, Guangzhou, GD 510640, P.R. China; 2 Computer School, South China Normal University, Guangzhou, GD 510631, P.R. China; E-Mail: zjq@scnu.edu.cn

**Keywords:** Enhanced Thematic Mapper plus (ETM+), Surface urban heat island (SUHI), Land surface temperature (LST), Variance segmenting, Thermal infrared image, Hot island area

## Abstract

Ten cities with different population and urban sizes located in the Pearl River Delta, Guangdong Province, P.R. China were selected to study the relationships between the spatial extent of surface urban heat islands (SUHI) and five urban characteristic factors such as urban size, development area, water proportion, mean NDVI (Normalized Vegetation Index) and population density, *etc.* The spatial extent of SUHI was quantified by using the hot island area (HIA). All the cities are almost at the same latitude, showing similar climate and solar radiation, the influence of which could thus be eliminated during our computation and comparative study. The land surface temperatures (LST) were retrieved from the data of Landsat 7 Enhanced Thematic Mapper Plus (ETM+) band 6 using a mono-window algorithm. A variance-segmenting method was proposed to compute HIA for each city from the retrieved LST. Factors like urban size, development area and water proportion were extracted directly from the classification images of the same ETM+ data and the population density factor is from the official census. Correlation and regression analyses were performed to study the relationships between the HIA and the related factors, and the results show that HIA is highly correlated to urban size (r=0.95), population density (r=0.97) and development area (r=0.83) in this area. It was also proved that a weak negative correlation existed between HIA and both mean NDVI and water proportion for each city. Linear functions between HIA and its related factors were established, respectively. The HIA can reflect the spatial extent and magnitude of the surface urban heat island effect, and can be used as reference in the urban planning.

## 1. Introduction

Land surface temperature (LST) has a direct impact on air temperature and it is also one of the key parameters in the physics of land-surface processes on regional and global scales. As a phenomenon modifying regional microclimate, urban heat island (UHI) effect has been studied for a long time. UHI can be described for different surfaces based on different methods and air temperature is widely used to measuring UHI. Two types of UHI are generally classified such as canopy layer (UCL) heat island and the boundary layer (UBL) heat island [1]. Voogt and Oke gave a detailed description about the UHIs for different surfaces and pointed out that thermal remote sensors could be used to observe the surface urban heat island (SUHI) [2].

Traditionally, temperature data for UHI study are mainly collected from weather stations or gathered along traverses with thermometers mounted on automobiles, which proves to be difficult to acquire the detailed spatial distribution of the temperature due to the limited locations for temperature measurement. Comparatively, thermal remote sensing technology can acquire LST distribution over the entire urban area, which has been used to study SUHI widely. Streutker studied the growth of SUHI of Houston in a 12-year's interval using the infrared channels of the Advanced Very High Resolution Radiometer (AVHRR) [3]. Weng performed a fractal analysis of SUHI of the city of Guangzhou by using Landsat TM data for the years of 1989, 1996 and 1997 [4]. Some other researchers investigated the impact of SUHI and found many related factors that affected the SUHI, such as the vegetation abundance, soil moisture and roughness properties of the land surface [5-7]. Weng investigated the impact of urban expansion on surface temperature in the Pearl River Delta, China and turned out several constructive suggestions concerning management of the adverse effects of urban development [8]. Lo, Quattrochi and Luvall used high-resolution thermal infrared remote sensing and GIS to assess the urban heat island effect [6]. It is clear that the UHI effect is different at different seasons or even at different time of a day. Klysik and Fortuniak found that the greatest differences of UHI occurred during summer nights when the sky was clear [9]. In the study of [10], the maximum temperature differences were measured in the early noon hours while the minimal temperatures were observed just before sunrise. In some studies, much attention has been paid to the relationship between land surface temperature and vegetation abundance [11-13]. Oke measured the urban heat islands based on air temperature data for ten settlements in the St. Lawrence Lowlands and demonstrated that the heat island intensity under cloudless skies was related to the inverse of the regional wind speed, and the logarithm of the population [14]. Streutker studied the urban-rural temperature differences by modeling the urban heat island as a two-dimensional Gaussian surface superimposed on a planar rural background using surface temperature maps derived from satellite sensed data [15]. Zhang and Wang studied LST changes based on robust statistics in Pearl River area after calculating the urban compactness by the method proposed by Zhang and Wang [16-18]. García-Cueto *et al.* used the data of air temperature and surface temperature retrieving from both NOAA's AVHRR and Landsat thermal images to study the relationship between UHI and land use at different spatial and temporal resolutions [19]. Lougeay *et al.* studied the intra-urban temperature patterns and associated land covers on the other hand [20].

In the paper of [19], two associated processes that affect UHI are pointed out: the first one is the alteration of land cover during the urbanization, which will inevitably result in the modification of the surface atmospheric energy balance; the second one is the activities that take place in the cities, which generate waste energy that contribute to the urban heating. In this paper, we study the relationship between UHI and land cover factors on the other hand, and try to explore a method to compute the spatial extent of SUHI in ten cities, namely the hot island area (HIA), directly from ETM+ data and study the relationships between HIA and five urban characteristic factors, i.e., urban size, development area and water proportion extracted directly from the classification images derived from the same ETM+ data, vegetation abundance expressed by mean NDVI and population density from the official census. As the UHI has important implications for human comfort and health, urban air pollution, energy management, and urban planning, the study for the quantified relationship between SUHI and related factors will be a suitable approach for the city administrators to conduct the urban planning.

It has long been known that strong correlations exist between the traditional UHI air temperature metric (Δ T u - r) and urban-extent indicators such as population [1, 21]. LST however is quite different from meteorological station air temperatures and so, in this paper, we wish to correlate statistics on LST with urban extent. Thermal remote sensing image has been used to express the UHI; however, the high LST area cannot be regarded as a high UHI area due to the strong heterogeneities of the urban land. It is necessary to circle the UHI area in the image and the determination of the threshold is critical. The proposed method in this study can be used to extract the HIA of a single city as well as to compare HIA of multiple cities. And the surface temperature in this paper was used in different way from the traditional study, to extract HIA on the basis of robust statistical method beyond measuring the intensity of UHI. This is another object of this study.

## 2. Study area

**Figure 1.**
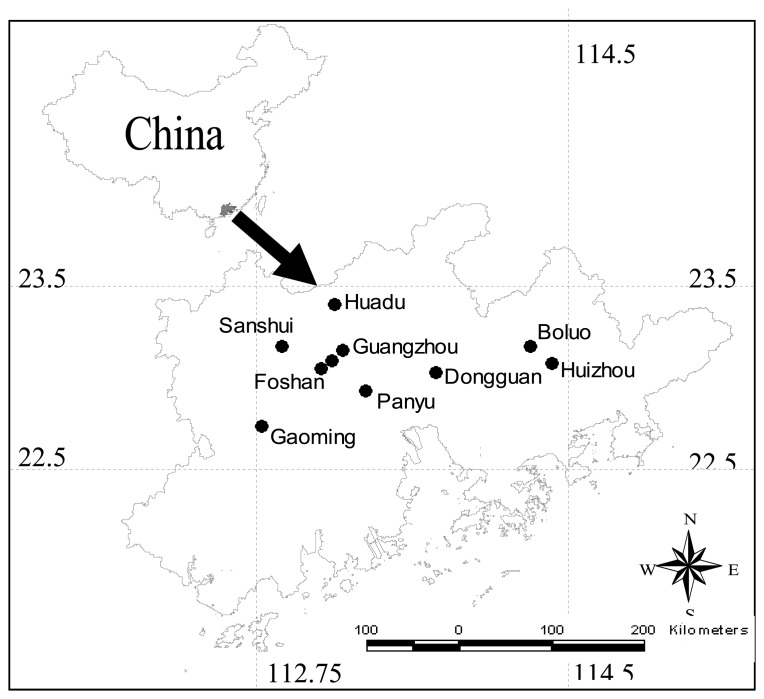
Map of the Pearl River Delta, showing the ten cities selected for the study.

Ten cities selected for this study are Guangzhou, Boluo, Dongguan, Panyu, Foshan, Gaoming, Huadu, Huizhou, Nanhai and Sanshui in Guangdong Province, China. They are in one scene of Landsat 7 Enhanced Thematic Mapper Plus (ETM+) image (Path/Row: 122/44) and situated around the same latitude. The whole study area (22.5- 23.5°N, 112.75-114.5°E) lies in the Pearl River Delta and enjoys a subtropical climate (Figure 1). The average annual temperature is between 18.7 and 23.4 °C and the average annual rainfall reaches 1,500 to 2,000 millimeters. Guangzhou, with the total area of over 7000 square kilometers and the population of more than seven millions, is the largest city in the study area [22]. With only 270 persons per square kilometer, Boluo has the lowest population density, while Guangzhou has the highest population density among then cities, reaching 17282 persons per square (Table 1).

The study area has experienced great changes in the past 20 years and undergone a quick urbanization process since 1978 when the economic reform campaign initiated [23-25]. With the quick progress of the economy, especially the rapid industrialization, a great amount of agriculture land has been converted into construction area and development sites. According to the previous study, more than 21,000 km^2^ of the cropland was lost in a single city (Dongguan) from 1988 to 1993 [24, 26-27]. The population and urban size have grown tremendously and the UHI effect in this area has become more serious in the study area. The rapid urbanization drive in the Pearl River Delta resulted in a city group characterized by different urban sizes, different population densities and different vegetation abundances *etc.*, which makes it a very typical area for studying the UHI effect and the relationships between spatial extent of the UHI and some urban characteristic factors such as urban size and vegetation abundance. Particularly, these cities are located in the same ETM+ scene and have very similar latitudes and climates, which can help to eliminate the uncertainties for retrieving surface temperature from the ETM+ data.

## 3. Materials and methods

### 3.1. Image and Pre-processing

One scene of Landsat 7 ETM+ image, cloud-free and dated on January 17, 2003, around 10:30 a.m. local time was acquired from China Remote Sensing Satellite Ground Station in Beijing (detailed information is available at http://www.rsgs.ac.cn). A systematically geometric and radiometric correction were performed to the image data using the calibration parameter file (CPF) released by the Earth Resources Observation Systems (EROS) Data Center (EDC), USGS before the satellite image was delivered, and the quality of Landsat image was in 1B level. The Landsat image, including the thermal band, was further rectified to Universal Transverse Mercator coordinate system and was re-sampled using the nearest neighbor algorithm with a pixel size of 30 by 30 m for all bands and the resultant root mean square error (RMSE) less than 0.5 pixels. The air temperature and moisture data were collected from the weather stations distributed in the ten cities as references for retrieving land surface temperature from the thermal remote sensing image.

### 3.2. Estimation of Vegetation Abundance

NDVI is commonly used as an index to show the vegetation abundance. Fung and Siu used NDVI to monitor the environment quality and its changes, and Gallo and Tarpley used NDVI to study the relationship between vegetation abundance and thermal remote sensing surface temperature [28-29]. Weng *et al.* used vegetation fraction derived from a spectral mixture model as an alternative indicator of vegetation abundance to study its relationship with LST [11]. Huete used the factor of soil-adjusted vegetation index (SAVI) to correct the impact of soil reflectance on NDVI [30]. In this study, we used the NDVI data computed from the red (0.63-0.69μm) and near-infrared (0.76-0.90μm) bands of the ETM+ image to estimate the vegetation abundance and study the relationship between the urban mean NDVI and the spatial extent of SUHI expressed by HIA. The urban mean NDVI is the average NDVI value of urban pixels, expressed by the ratio of sum of the NDVI value and the total pixel numbers of the city. NDVI for Landsat ETM+ images is calculated through:

(1) $$\text{NDVI}=\frac{TM_{4}-TM_{3}}{TM_{4}+TM_{3}}$$

### 3.3. Factors Extracted from Classification Images

Among these 5 factors chosen for this study, except that the population density was drawn from the official census, all the other 4 factors were derived from the ETM+ data. The urban size, development area and water proportion were extracted directly from the classification images. Here the urban size and development area of each city can be easily calculated from the sum of corresponding land use/cover pixels in the classification images, while the water proportion is the ratio of water area again the total area of urban area (including both land and water areas), and can be computed by the following equation:

(2) $$P=\overset{S_{\text{water}}}{\;}/\underset{\left(S_{\text{water}}+S_{\text{urban}}\right)}{\;}$$

where *P* is the water proportion, *S_water_* is the pixel area of water; *S_urban_* is the pixel area of urban-used land. The values of these factors extracted from the classification image and mean NDVI are listed in Table 1.

### 3.4. Estimation of Ground Surface Emissivity

Ground surface emissivity is very critical for determining the surface temperature, but it is particularly difficult to measure for the influence of a variety of factors such as ground wetness, structure and roughness, *etc.* For the Landsat ETM+ data, one pixel covers an area of 30 by 30 m on the ground, which is probably contain multiple land covers. Therefore, the emissivity from a pixel is determined by land objects and their emitting directions [31]. Different techniques have been designed to estimate the emissivities of ground objects, separate temperatures from emissivities and mitigate the effect of emissivity on estimated LST [32-34]. In some studies, ground surface emissivity was estimated by NDVI value and the land surface was divided into vegetated and non-vegetated areas with a threshold established to the NDVI image, so that corrections for emissivity can be performed [5, 11].

According to the study of [35], the relationship between emissivity and NDVI can be expressed by the following equation:

(3) ɛ=1.0094+0.047*ln(NDVI)

when the NDVI value ranges from 0.157 to 0.727.

Valor and Caselles [36] developed another method to calculated emissivities from NDVI values according to the following equation:

(4) $$ɛ={ɛ}_{v}P_{v}+{ɛ}_{g}(1-P_{v})+4<dɛ>P_{v}(1-P_{v})$$

where *ε* is ground emissivity; *ε_v_* is emissivity of full vegetation cover area; *ε_g_* is emissivity of bare ground area; <*dε*> is revised parameter and averagely value 0.01; *P*_v_ stands for the percentage of vegetation abundance in a pixel. Usually, the *P_v_* can be determined by the following equation:

(5) $$P_{v}=\frac{\text{NDVI}-{\text{NDVI}}_{s}}{{\text{NDVI}}_{g}-{\text{NDVI}}_{s}}$$

where *NDVI_g_* is *NDVI* value of full vegetation cover area and *NDVI_s_* is the *NDVI* value of bare ground. In the study of Valor and Caselles, it was also pointed out that the relationship between emissivity and *NDVI* is complex and the emissivities calculated by *NDVI* are just approximate values. After comparing two methods of calculating emissivity from *NDVI*, Valor and Caselles's method was found more suitable, which was chosen to retrieve the ground emissivities from *NDVI* in this study.

### 3.5. Retrieving of Land Surface Temperature

The signals received by the thermal sensors can be converted to at-sensor radiance (*L_λ_*). Radiance values from the ETM+ thermal band and then be transformed to radiant surface temperature, namely brightness temperature, according to Eq. (6) using thermal calibration constants supplied by the Landsat Project Science Office [37]:

(6) $$T_{s}=\frac{K_{2}}{\ln(\frac{K_{1}}{L_{\lambda }}+1)}$$

where *T_s_* is the effective at-satellite temperature in K, *K_1_* and *K_2_* are the pre-launch calibration constants (For Landsat 7 ETM+: *K_1_*=666.09 W/(m^2^·sr·*μm*) and *K_2_*=1282.71 K).

The temperature calculated by Eq. (6) is not the actual LST, but the at-sensor brightness temperature. To obtain a reasonably high quality of LST, four stages of correction process are required: (1) spectral radiance conversion to at-sensor brightness temperature; (2) correction for atmospheric absorption and re-emission; (3) correction for surface emissivity; and (4) correction for surface roughness [2]. Traditionally, the retrieval of LST from Landsat TM6/ETM+6 was mainly completed through the method of atmospheric corrections. The principle of atmospheric corrections is to subtract the upward atmospheric thermal radiance and the reflected atmospheric radiance from the observed radiance at satellite level so that the brightness temperature at ground level can be directly computed [38-39]. Several programs such as LOWTRAN, MODTRAN and 6S have been designed for the atmospheric corrections, but the atmospheric corrections therefore prove to be difficult to complete because of many necessary parameters, and this is especially the case when the corrections have to be performed for the image at the time when the satellite passed. Base on the thermal radiance transfer equation, a mono-window algorithm for retrieving LST from thermal band of Landsat TM and ETM+ data was designed and only three parameters were required for the algorithm: emissivity, transmittance and effective mean atmospheric temperature [38-39]. Being used to calculate the land surface temperature in this paper, the mono-window algorithm was expressed as follows:

(7) $$T_{s}=\frac{a_{6}(1-C_{6}-D_{6})+[b_{6}(1-C_{6}-D_{6})+C_{6}+D_{6}]T_{6}-D_{6}T_{a}}{C_{6}}$$

where T_s_ is the land surface temperature in K, *T_6_* is the brightness temperature computed from Eq. (6) *T_a_* is the effective mean atmospheric temperature in K; *a_6_* and *b_6_* are constants with values of – 67.355351 for *a_6_* and 0.458606 for *b_6_* when the LST is between 0-70 ^0^C [38-39]. *C_6_* and *D_6_* can be calculated by the following equations:

(8) $$C_{6}={ɛ}_{6}\tau_{6}$$

(9) $$D_{6}=(1-\tau_{6})[1+(1-{ɛ}_{6})\tau_{6}]$$

where *ε_6_* is the ground surface emissivity and *τ_6_* is the atmospheric transmittance. *T_a_, ε_6_, τ_6_* are three parameters needed to covert the brightness temperature to LST. Atmospheric transmittance (*τ_6_*) could be estimated according to the near-surface air temperature and the water vapor data from the local meteorological observatories because there exists a linear relationship between *τ_6_* and water vapor. The effective mean atmospheric temperature (*T_a_*) was calculated by the linear equations corresponding to the four standard atmospheres (Eq. (10)):

(10) $$\begin{array}{ll} T_{a}=25.9396+0.88045T_{0}\;(\text{For USA}1976) & \left(a\right)\\ T_{a}=17.9769+0.91715T_{0}\;(\text{For tropical}) & \left(b\right)\\ T_{a}=16.0110+0.92621T_{0}\;(\text{For mid}-\text{latitude summer}) & \left(c\right)\\ T_{a}=19.2704+0.91118T_{0}\;(\text{For mid}-\text{latitude winter}) & \left(d\right) \end{array}$$

where *T_a_* is the effective mean atmospheric temperature in K, *T_0_* is the near-surface air temperature in K; both of them could be acquired from the local meteorological observatories. The four standard atmospheres were provided by atmospheric simulation model LOWTRAN 7 and more detailed information about the linear equations could be found in the paper written by Qin *et al* (2001) [38]. In this paper, the second equation (Eq. (10b)) was used.

### 3.6. Method to Calculate The HIA

Most previous studies are focused on the spatial distribution or temporal changes by equally segmenting the urban surface temperatures from thermal remote sensing images, however, this equally segmenting method is not suitable when threshold values are selected arbitrarily, and the results may not well represent the high-temperature area. In this study, a standard deviation segmenting method was proposed to calculate the HIA from the LST image for seeking a more suitable threshold value.

**Figure 2.**
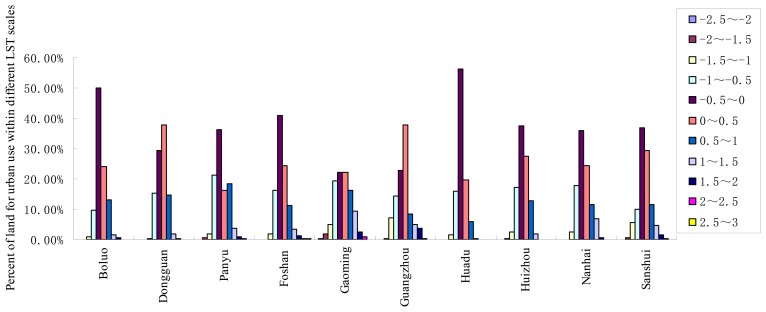
Percent of urban-used land within different LST scales for the ten cities in Guangdong.

With standard deviation segmenting method, both the hot island and cold island can be extracted by determining a threshold from the standard deviation of the surface mean temperature for each city. In order to calculate the HIA, five steps maybe needed as following:

Step 1. Calculate the mean surface temperatures for the cities and their standard deviation.
Step 2. Use the following equation to calculate the temperature threshold values.
  (11) T=α±χ*sd
  where *T* stands for the temperature threshold value, *a* is the mean value of the surface temperature for each city, *χ* (*χ* = -2.5, -2, -1.5, -1, -0.5, 0.5, 1, 1.5, 2, 2.5, 3) is the times of standard deviation, while *sd* is simply the standard deviation. Here eleven values were prepared for *χ* and eleven temperature thresholds were calculated according to the different values of *χ* ranging from −2.5 to 3 by the interval of 0.5.
Step 3. Divide the surface temperature into eleven scales according to the threshold values calculated in the above step.
Step 4. Calculate the percentages of urban pixels in different surface temperature scales and their distribution in each city is plotted in Figure 2.

It can be found from Figure 2 that the urban LST follows the Gaussian distribution mode and most of the urban pixels (>80%) are within the scale of ±1 times of the standard deviation. As a result, the mean surface temperature ± 1 times of standard deviation can be determined as the background value for urban LST according to the robust statistical rule, and can thus be recognized as the threshold value for extracting the hot or cold island.

The last step is to calculate the HIA. The threshold of (*a* + *sd*) was used to extract the outlines of the hot island and then determine the HIA by calculating the total number of pixels that the temperature is higher than (*a* + *sd*). HIA could reflect the spatial extent and the seriousness of SUHI and could be used to quantify the SUHI effect. The calculated HIA of each city are listed in Table 2, from which, it can be discovered that Guangzhou shows the largest spatial extent with the HIA value of 20.05Km^2^, while the smallest value (0.58Km^2^) of HIA was found at Boluo, which is a small city with a population of 25,000 and the location of the eastern part of the Pearl River Delta. It should be emphasized that this standard deviation segmenting method was based on the statistic knowledge and was more suitable to extract the hot island compared with the density equally-slicing method.

## 4. Results and Discussions

### 4.1. Retrieved LST of Each City and Error Analysis

**Figure 3.**
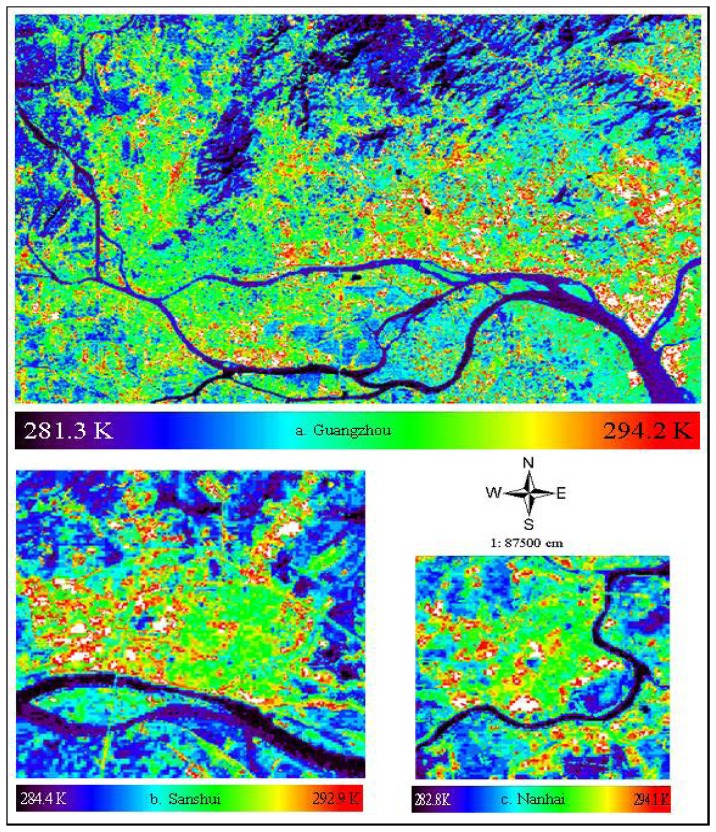
LST images retrieved from ETM Band 6 of three cities in Guangdong.

The LSTs of ten cities selected for this study were retrieved from ETM+ Band 6 by Eq. (7) and the LST images for three cities with different sizes are shown in Figure 3 as examples. The statistical results of surface mean temperature of urban-used land in each city are listed in Table 2. As shown in *Figure* 3, the LST ranges from 281.3 to 294.2 K for Guangzhou, from 284.4 to 292.9 K for Sanshui and from 282.8 to 294.1 K for Nanhai, respectively. The LST ranges of other cities can be found in Table 2. Generally, the surface temperature fluctuates between 281.3 K and 294.9 K at the time of the day. Both the minimum (281.3 K) and maximum surface temperatures (294.9 K) in 10 cities were found in Guangzhou, which has the largest population and urban area among all these cities.

The best method to evaluate the accuracy of the LST is to compare it with the actual surface temperature measured at the time when the satellite passed, which proves very difficult to be acquired. For the method used to derive the LST, there are two types of errors: the absolute error and the relative one. The absolute error arisen from the computation process and was found to range approximately from 0.2-0.3 K [38-39]. The relative errors were mainly due to the three aspects: (1) the estimation of atmosphere transmittance, (2) the estimation of emissivities of different land covers and the precision of classification, and (3) the estimation of effective mean atmospheric temperature. The precision can be quantified by the following equation:

(12) $$\delta T_{s}=\left|T_{s}(\chi +\delta_{\chi }){-\text{T}}_{s}(\chi )\right|$$

where δT_s_ is the error of temperature, δ_χ_ is the error from estimating parameter *χ*, *T_s_(χ* + *δ_χ_)*, and *T_s_(χ)* are the temperatures computed from Eq. (7) using the data of *(χ* + *δ_χ_)* and *χ*. Results demonstrate that the relative errors for ten cities of this study are 1.2 K on average.

### 4.2. Correlation Analysis between HIA and 5 Factors

Firstly, we performed a correlation analysis and the results are listed in Table 3. The coefficients of correlation demonstrate that the HIA is highly correlated to the urban size, population density and development area. These three factors are all positively correlated with the HIA, therefore demonstrating remarkable influences on the latter. Although the negative relationships found between HIA and water proportion or urban mean NDVI are not very significant, the impacts posed by water proportion and urban mean NDVI on the SUHI effect of a city are still very strong due to their high thermal capacity and large proportion values. Obviously, water area and vegetation abundance should be well preserved and be greatly increased for an effective control of the SUHI effect.

### 4.3. Regression Analysis between HIA and 5 Factors

**Figure 4.**
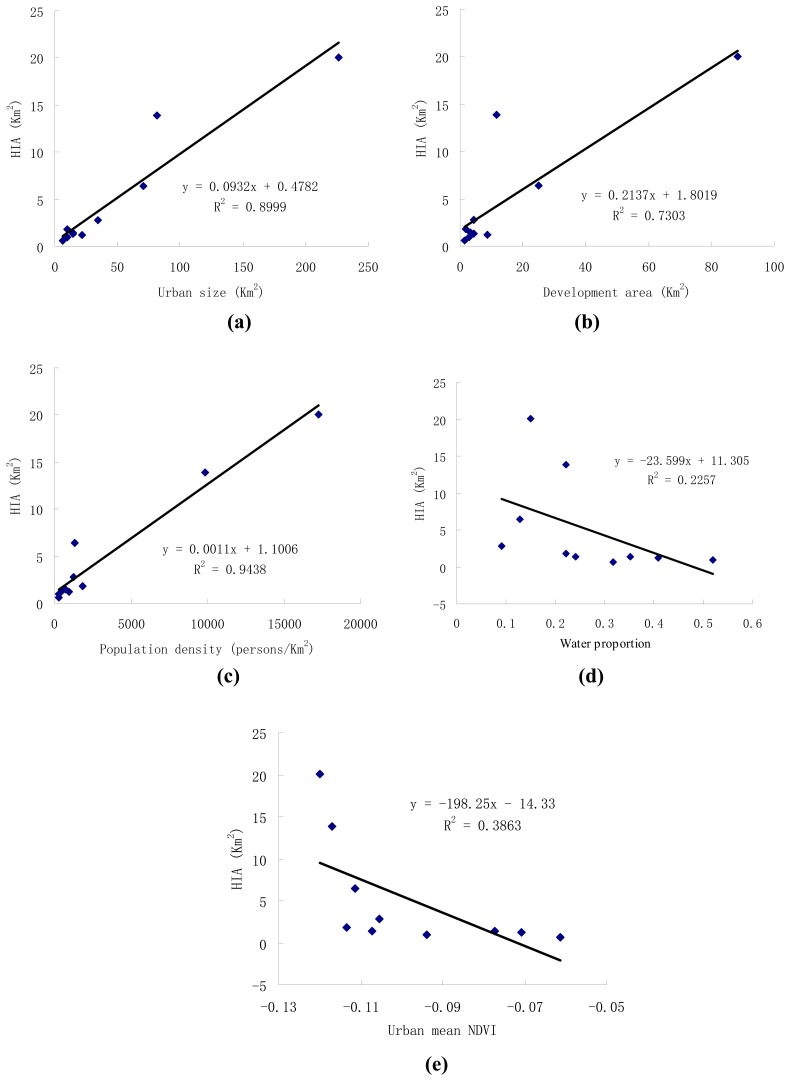
**(a)** Relationship between HIA and urban size in 10 cities in Guangdong, China. **(b)** Relationship between HIA and development area in 10 cities in Guangdong, China. **(c)** Relationship between HIA and urban population density in 10 cities in Guangdong, China. **(d)** Relationship between HIA and water proportion in ten cities in Guangdong, China. **(e)** Relationship between HIA and mean NDVI value in ten cities in Guangdong, China.

The SUHI effect is a phenomenon resulted from urbanization. The characteristics of a city such as the city size, population density, water and vegetation abundances have a direct and obvious influence on the SUHI. Consequently, it is critically important to research the relationships between the SUHI effects and the associated city characteristics. In this section, the regression analysis between the HIA and these factors were conducted, and the results were plotted in Figure 4.

From the results showed from Figure 4, it can be found that the regression functions between HIA and urban size, population density are very significant, and both the R square values are as high as 0.9. Development area is also an important factor affecting the SUHI effect in this area. Development area mainly refers to those construction sites and it is very common in this area for the rapid economic development and quick urban sprawl. By comparing the slope of the three regression functions, it can be found that the growth of the development area has the most distinct relationship with HIA. As far as the correlation analysis is concerned, the coefficient with urban size is the greatest.

Water proportion and urban mean NDVI are all associated with the properties of ground surface in the urban regions, and they will both influence the surface temperature significantly. Here water proportion and mean NDVI were used to represent the quantities of water and vegetation of a city. Their relationships with the HIA are plotted in Figure 4 (d) and (e). The regression analysis was also performed, but the R square values for both water proportion (r^2^=0.23) and mean NDVI value (r^2^=0.39) are very small, demonstrating weak relationships between the HIA and these two factors.

From the above analysis, it can be found that three factors: urban size, population density and development area are positively relevant with HIA. For further understanding the control of these factors to HIA, a multiple linear regression model based on these three significant factors was built and the regression equation was as follows.

(13) $$\text{HIA}=0.183*\text{urban}-\text{size}-0.265*\text{development}-\text{area}-0.304(r^{2}=0.998)$$

The R square value of the model is as much as 0.998 and the test value for the coefficients of urban size and development area are smaller than 0.001. Though the population density is an important factor, test value shows that it is not significant in the equation, so the multiple-regression model contains only two factors.

## 5. Conclusions

The surface temperature is of prime importance to the study of urban climatology. The effect of SUHI is affected by many urban characteristic factors, such as water proportion, roughness of ground surface, urban size, urban population density and vegetation abundance, *etc.* In this paper, the land surface temperatures (LST) were retrieved from the data of Landsat 7 Enhanced Thematic Mapper Plus (ETM+) Band 6 using a mono-window algorithm. The error analysis indicates that the precision of this algorithm is less than 1.2 K, which proves to be sufficient for studying the regional SUHI. A standard deviation segmenting method was proposed to retrieve the outline of hot island in each city, and the results demonstrated that this method could be used to extract the hot island area directly from the LST images and to describe the spatial extent of the SUHI.

In this study, five urban characteristic factors were selected (four of them were calculated directly from the ETM+ images and one of them was calculated from the official census), and their relationships with the HIA were analyzed. Results of regression analysis performed for the 10 cities show that the HIA is highly correlated to the urban size (r=0.90), population density (r=0.94) and development area (r=0.73) of a city, and a weak negative relationship exists between the HIA and the mean NDVI or water proportion for the ten cities in Guangdong. Linear functions describing the relationships between the HIA and the five related factors were established respectively, and a multiple linear regression model was constructed.

The SUHI effect is a product of urbanization. The urban size is one of the most distinct characteristics for a city, and the effect posed by urban size on SUHI will be worth further study. Additionally, urban population also plays a key role on SUHI.

Some uncertainties still remain when retrieving LST from the Landsat 7 ETM+ Band 6, and they are mainly due to the effect of the atmosphere. The effect of SUHI was affected by many factors, but only five factors were selected for this study. Therefore, further studies should be continued and some other factors, taking the roughness of ground surface for example, in a city should be taken into account.

## Figures and Tables

**Table 1.** Factors extracted from the classification image and mean NDVI ^1)^.

City name	Population density (Persons / km^2^)	Urban size (Km^2^)	Urban mean NDVI value	Water proportion	Development area (Km^2^)
Boluo	270	6.01	-0.0613	0.3167	2.37
Dongguan	1326	71.10	-0.1115	0.1283	25.07
Panyu	1242	24.67	-0.1054	0.0920	4.38
Foshan	9815	81.75	-0.1168	0.2207	8.14
Gaoming	315	9.60	-0.0937	0.6614	3.08
Guangzho	17282	226.76	-0.1089	0.1507	88.28
Huadu	742	16.23	-0.1072	0.2422	3.22
Huizhou	955	21.40	-0.0707	0.4090	8.79
Nanhai	1854	10.49	-0.1134	0.2210	0.91
Sanshui	542	14.58	-0.0773	0.3514	4.20

1) The population data are drawn from Report of the Fifth Census of China (2000).

**Table 2.** The statistical results of surface temperature for the ten cities.

City name	Minimum temperature (K)	Maximum temperature (K)	Mean temperature (K)	Standard deviation	Variance	HIA (Km^2^)
Boluo	283.3	292.6	288.9	0.974	0.948	0.58
Dongguan	284.2	294	289.1	1.303	1.699	6.46
Panyu	283.1	294.8	288.8	1.564	2.447	2.77
Foshan	281.7	294.1	288.4	1.687	2.845	13.91
Gaoming	284.7	292.6	288.0	1.255	1.575	1.00
Guangzhou	281.3	294.2	288.4	1.454	2.115	20.05
Huadu	282.5	291.7	287.8	1.145	1.312	1.44
Huizhou	283.2	292.4	288.3	1.159	1.344	1.18
Nanhai Sanshui	282.8 284.4	294.1 292.9	289.0 288.2	1.654 1.371	2.737 1.881	1.76 1.32

**Table 3.** Results of correlation analysis between HIA and five factors for ten cities in Guangdong.

Factors	Coefficient of correlation with HIA	P-value for T-test
Urban size	0.950	0.000
Population density	0.971	0.000
Water proportion	-0.418	0.206
Urban mean NDVI value	-0.515	0.128
Development area	0.833	0.003
